# Risk Factors for Low Birthweight in Zimbabwean Women: A Secondary Data Analysis

**DOI:** 10.1371/journal.pone.0129705

**Published:** 2015-06-26

**Authors:** Shingairai A. Feresu, Siobán D. Harlow, Godfrey B. Woelk

**Affiliations:** 1 University of Pretoria, School of Health Systems and Public Health, Epidemiology and Biostatistics, Pretoria, South Africa; 2 University of Zimbabwe, Department of Community Medicine, Harare, Zimbabwe; 3 School of Health Sciences, College of Health Sciences, Walden University, Minneapolis, Minnesota, United States of America; 4 Department of Epidemiology, School of Public Health, University of Michigan, Ann Arbor Michigan, United States of America; 5 Elizabeth Glacier AIDS Foundation, Washington, D.C., United States of America; UCL Institute of Child Health, University College London, UNITED KINGDOM

## Abstract

**Background:**

Low birth weight (LBW) remains the main cause of mortality and morbidity in infants, and a problem in the care of pregnant women world-wide particularly in developing countries. The purpose of this study was to describe the socio-demographic, nutritional, reproductive, medical and obstetrical risk factors for delivering a live LBW infant at Harare Maternity Hospital, Zimbabwe.

**Methods:**

A secondary data analysis from data obtained through a questionnaire and delivery records was conducted. Linear regression models with a complimentary log-log link function were used to estimate the relative risks for all LBW, term LBW and preterm LBW.

**Results:**

The frequency of LBW was 16.7%. Lack of prenatal care (adjusted relative risk [ARR] 1.69, 95% CI 1.44, 1.98), mother’s mid-arm circumference below 28.5 cm, (ARR 1.35, 95% CI 1.19, 1.54) and rural residence (ARR 1.22, 95% CI 1.04, 1.40) increased the risk of LBW. Eclampsia, anemia, and ante-partum hemorrhage, were associated with LBW (ARR 2.64, 95% CI 1.30, 5.35; ARR = 2.63, 95% CI 1.16, 5.97; and ARR = 2.39, 95% CI 1.55, 3.68), respectively. Malaria increased the risk of LBW (ARR = 1.89, 95% CI 1.21, 2.96). Prenatal care, infant sex, anemia, antepartum hemorrhage, premature rapture of membranes and preterm labor were associated with the three LBW categories. History of abortion or stillbirth, history of LBW, malaria, eclampsia, and placenta Previa, were associated with all LBW and preterm LBW, while pregnancy induced hypertension, and number of children alive were associated with all LBW and term LBW.

**Conclusions:**

LBW frequency remains high and is associated with nutritive, reproductive, medical and obstetrical factors. Preterm LBW and term LBW have similar and also different risk factors. Understanding the role of different risk factors in these different LBW categories is important if the goal is to reduce LBW frequency, and its complications, in Zimbabwe.

## Introduction

Low birth weight (LBW) defined as a birth weighing 500 grams and above but below 2,500 grams irrespective of gestational age, continues to be a problem world-wide [1–4] and a cause of infant mortality [1, 5–7], but more in developing countries [8–14]. More than 20 million infants in the world (15.5% of all births) are born with LBW [1, 5]. Ninety-five percent of LBW births are in developing countries with the rate of LBW in developing countries being more than double that of developed countries (16.5% and 7.0%, respectively) [1]. In Sub-Saharan Africa, the rate of LBW was around 15.0% [1]. The incidence of LBW in a few selected countries in Africa range between 7.0% and 18.0%, and is comparable to between 9.0% and 17.9% for Latin America, and between 5.2% and 30.0% for Asia [5]. These levels are high, compared to developed country rates, which in Europe range between 4.0% and 7.0% and in North America from 6.0% to 6.9% [5].

LBW can be a consequence of preterm birth (PTD), which is birth before 37 completed weeks of pregnancy, or fetal growth restriction or intrauterine growth restriction (IUGR), which means that a baby doesn’t gain the appropriate weight before birth [3, 8–10, 12, 15]. The earlier the baby is born, the lower the birth weight, but babies may be small simply because their parents are [8–10]. At the extreme end of LBW a distinction is made of very LBW (VLBW), depicting infants less than 1,500 g and extremely LBW (ELBW), depicting infants less than 1,000 g [16].

LBW is a problem of maternity care in Zimbabwe, with prevalence ranging from 10.8% to 24.3% [1, 9–12, 17]. Although LBW has been well studied in developed countries [3, 8], data from developing countries such as Zimbabwe has been previously limited, but has steadily somewhat improved over the years [8–12, 18–19], particularly as LBW relates to HIV infection [19–23]. Studies have not adequately addressed risk factors for LBW, or differentiated LBW from preterm birth, but in our previous studies [10–11], we described limited risk factors of preterm and term LBW from annual delivery logs. Unlike this present study where our focus is on LBW, in our previous study, using the same dataset, we had limited our analysis to preterm birth irrespective of birth weight [24]. But, there is still a need to describe risk factors for LBW in Zimbabwe from these data. This study is among the few to assess risk factors for LBW in Zimbabwe. Our objective is to describe the socio-demographic, nutritional, reproductive, medical and obstetrical risk factors of LBW among live births in a 3-month period of study at Harare Maternity Hospital.

## Materials and Methods

In this paper we report findings from a secondary analysis of data from a study which primarily assessed risk factors for preterm birth, conducted among women delivering at Harare Maternity Hospital between March and June 1999. While the data is from 1999, apart from the role of HIV/AIDS, prevalence of LBW remain the same at 11.0% [4, 17, 21], also, the problem of limited data for this important problem of natality in Zimbabwe persists [4, 17]. The methods have been described elsewhere [24]. Briefly, all women delivering a singleton infant that survived the first hour of life were eligible to participate. For the 3,722 women who delivered during the study period, 527 (14.2%) were ineligible including 198 (5.3%) multiple pregnancies, 26 (0.7%) very ill babies 248 (16.7%) stillbirths and 52 (1.4%) early neonatal deaths. Of the 3,195 eligible women, 20 (0.6%) refused to participate, 27 (0.8%) could not be interviewed before discharge, 30 (0.9%) had incomplete records, and 8 (0.2%) did not have information on birth weight, leaving 3,110 (97.3%) eligible women for the LBW analysis.

In this present day analysis, the main outcome of interest was LBW, which was later categorized into term LBW, and preterm LBW infants, based on information collected from the medical records. Women were identified as having delivered a singleton LBW live infant if the infant weighed 500 grams, but below 2,500 grams at birth, irrespective of gestational age. Women who delivered a”normal weight, term” infant were all women who delivered a live-born singleton baby weighing 2,500 grams and more at term (37 weeks of gestation and above). Term LBW infants were defined as term infants who weighed more than 500 grams, but below 2,500 grams. Preterm LBW infants were defined as preterm infants who weighed more than 500 grams, but below 2,500 grams.

Based on original study [24], each day at 8 am and 2 pm, a list of women was made from the delivery logbook. Eligible women who agreed to participate and signed the consent form, had their medical records abstracted, completed a short interview regarding demographic and lifestyle factors, and their baby was examined for maturity. Six research assistant were used for data collection, and to administer the Ballard method of assessing gestational age [25].

For socio-demographic factors, age of mother was calculated as the number of years from her date of birth to her previous birthday. Information of marital status (currently married or living as married, never married, separated, divorced or widowed), education of mother and father (less than primary education, having achieved primary education, secondary education and above), employment status of mother and father (yes/no), residence (urban/rural), if they had electricity (yes/no), had water supply (yes/no), and had a toilet (yes/no), were obtained by interview.

For lifestyle factors, information on alcohol drinking (chibuku, beer, spirits or wine), or drank home brew (mahewu), were obtained through the questionnaire. Mahewu is a local non-alcoholic nutritious beverage made from corn meal, rapoko or sorghum, soya beans and sugar [24]. Chibuku is a locally brewed alcoholic drink, which could also be nutritious, containing soya beans, and sugar apart from the intoxicants. Women were also asked individually (yes/no), if they drank mahewu, chibuku, beer, spirits or wine during pregnancy. Women were asked about smoking during pregnancy (yes/no).

For anthropometric measurements, weight and height at first prenatal care visit of the mother were collected from medical records. Body mass index (BMI) was calculated as weight of mother at first contact, in kilograms divided by height in meters squared. Mid-arm circumference (MUAC) was obtained by measuring the length between shoulder and the elbow with arm bend, with circumference measured at the midpoint [26]. MUAC was later categorized, using Jellife standards at cut off of more than 28.5 centimeters (cm), less denoting under nutrition [27].

Mothers were grouped into those who attended at least one visit of prenatal care during pregnancy or otherwise. Parity (0, 1 to 2 and more than 2 pregnancies), prior history of abortion (delivery before 20 weeks of gestation, or infant weighing less than 500 grams at birth), stillbirth or LBW birth (yes/no) sex of the infant, and infant birth weight were abstracted from obstetrical records [24]. Information on a diagnosis of chronic medical condition or obstetrical complication including diabetes, hypertension, anemia, pregnancy induced hypertension, eclampsia, cardiovascular disease, ante-partum hemorrhage, premature rapture of membranes (PROM), preterm labor (PTL) with current pregnancy and placenta previa, based on diagnosis by attending medical doctor, was abstracted from obstetrical records [24]. History of infections during pregnancy of malaria, urinary tract infection, syphilis or gonorrhea, were also obtained from obstetrical records.

### Ethics Statement

When one is carrying out research on humans in Zimbabwe, it is required to get approval from and register the project with the Medical Research Council of Zimbabwe, which in turn gets the Research Council of Zimbabwe’s approval. This process involves seeking permission from the Permanent Secretary of Health and from the departmental heads of any institutions defined in the proposal. The principal investigator was responsible for processing this approval. The University of Michigan Institutional Review Board permission was also obtained prior to this study. The medical record review did not use personal identities and we requested exempt status for that portion of the study. A consent form, translated into Shona, was signed by each individual study participant. The study therefore, was approved by the University of Michigan Institutional Review Board and the Medical Research Council of Zimbabwe and permissions were obtained from the Ministry of Health and Harare Central Hospital. This article is based on secondary analysis of these data, therefore exempt for IRB.

### Data Sharing Plan

We have submitted the dataset to **Figshare** in SPSS and SAS format; direct links:

Feresu, Shinga (2015): LBW data. figshare.
http://dx.doi.org/10.6084/m9.figshare.1348840 http://figshare.com/articles/LBW_data/1348840 Retrieved 14:10, May 11, 2015 (GMT)
Feresu, Shinga (2015): LBW SAS data. figshare.
http://dx.doi.org/10.6084/m9.figshare.1348839 Retrieved 14:02, May 11, 2015 (GMT)
http://figshare.com/articles/thirdphase_sas7bdat/1348839

### Statistical analysis

Since a complete population of live births over a 3-month period within the hospital was collected, we estimated the relative risks of LBW, and its sub-sets (as common in Reproductive and Perinatal Epidemiology). In univariable analysis, crude relative risks, 95 percent confidence intervals and chi-square tests were calculated from cross-tabulations with each pair of the outcome variables (all LBW, term LBW and preterm LBW) and each risk factor (determinants or exposures) to assess the association between each potential risk factor and either all LBW, term LBW, or preterm LBW using EPINFO version 7. We grouped risk factors into major subsets including, socio-demographic; anthropometric and nutritional factors; reproductive factors; medical and obstetrical complications; and infections, in our crude analysis.

We then fitted generalized linear regression models with a complimentary log-log link function, because of the nature of our data, to estimate the adjusted relative risks of all LBW for these risk factors [28–29]. We limited our adjusted analysis to all LBW infants as numbers for term LBW and preterm LBW were too small, and were giving unstable estimates. We built a model of socio-demographic, anthropometric and nutritional factors and reproductive factors, using a cut off of a p-value of 0.10 for variables to be included and also taking into account their contribution to the model, or if they were established potential confounders for this outcome. We also adjusted for whether the mother was referred to Harare Maternity Hospital or not. In the second multivariable model, we examined the risk associated with medical and obstetrical complications and for infections after adjustment for socio-demographic, reproductive factors, anthropometric and nutritional factors. We did not analyze data for VLBW (births below 1,500 grams in weight) and ELBW (births below 1,000 grams in weight) as the numbers were small, and gave unstable estimates. Data were analyzed using SAS version 9.3 (SAS Institute, Cary, NC).

## Results

### Demographic characteristics, Anthropometric and Nutritional factors

Among the 3,110 live births at Harare Maternity Hospital over the 3-month period 16.7% (n = 520) were LBW births, of which 60 (11.5%) were VLBW and 18 (3.5%) were ELBW. About 493 (15.9%) were preterm births, 199 (6.4%) were term LBW, 302 (9.7%) were preterm LBW, and only 191 (6.2%) were preterm without LBW.

Age of mother ranged from 13 to 49 years (mean = 24.4 years). Very few mothers (7.3%) were above 34 years old, in Table 1. About three quarters of the mothers had attained a secondary level of education and almost all women were married, the majority lived in urban areas. Very few women (13.4%) were employed, and 82.2% of fathers had some form of employment.

**Table 1:** Distribution of Low Birthweight among Live Births by Socio-Demographic Characteristics for 3,110 Deliveries^§^ at Harare Maternity Hospital, Zimbabwe.

		Total births		LBW births		Term LBW		Preterm LBW	
		n	%†	n	%†	n	%†	n	%†
**Characteristic**	**Total**	3110		520	16.7	199	6.4	302	9.7
***Socio-demographic factors***									
Mother’s age*	Below 20	647	21.1	137	21.3	44	8.6	67	12.4
	20 to 34	2199	71.6	383	17.5	143	7.9	207	11.0
	above 34	224	7.3	34	15.3	10	5.4	22	11.2
**Maternal education** **	Less than primary	140	4.6	23	17.0	7	6.5	17	14.5
	Primary	629	20.5	105	17.0	45	9.0	59	11.3
	Form II	686	22.4	126	18.6	48	8.9	75	13.1
	Form IV	1565	51.0	249	16.1	94	7.2	140	10.3
	Form VI and above	47	1.5	7	14.9	3	7.3	4	9.5
**Paternal education** ***	Less than primary	38	1.3	10	27.8	2	6.7	6	17.6
	Primary	221	7.6	36	17.7	10	5.9	26	13.9
	Form II	268	9.2	43	16.3	16	7.4	23	16.2
	Form IV	2148	73.8	355	16.8	143	8.2	202	11.0
	Form VI and above	234	8.1	37	15.9	15	7.5	17	8.3
**Marital status** ****	Current or living as married	2863	95.1	466	16.5	180	7.7	267	10.9
	Never married	71	2.3	15	22.1	4	8.0	10	17.9
	Separated, Divorced or Widowed	174	5.6	38	22.4	15	11.5	17	17.1
**Mother employed** *****	Yes	420	13.4	78	18.8	28	8.2	49	13.5
	No	2688	86.6	441	16.7	171	7.8	252	11.0
Father employed******	Yes	2538	82.2	423	16.9	162	7.8	246	11.3
	No	551	17.8	87	17.3	32	7.8	50	11.7
**Residence** *******	Urban	2725	88.0	447	16.6	166	7.5	266	11.4
	Rural	371	12.0	72	19.2	33	11.1	35	11.7
**Have electricity** ********	Yes	2247	72.3	371	16.8	136	7.4	220	11.4
	No	861	27.7	148	17.5	63	9.1	81	11.3
**Have water supply** ********	Yes	2724	87.6	456	17.0	173	7.8	266	11.4
	No	384	12.4	63	16.8	26	8.5	35	11.0
**Have a toilet** ********	Yes	2888	92.9	486	17.1	191	8.1	276	11.2
	No	220	7.1	33	15.3	8	4.7	25	13.4
***Anthropometric measurements***									
**Body Mass Index** *********	Less than 18.49	19	1.4	6	31.6	2	13.3	3	17.6
	18.49 to less than 25	631	45.8	99	16.0	44	8.2	47	8.6
	25 and above	726	53.8	60	8.4	23	3.7	60	6.0
**Mid-arm**	17 to 28.5 centimeters	1770	66.8	352	20.2	147	10.4	194	13.1
**circumference** **********	>28.5 to 43 centimeters	879	33.2	100	11/5	34	4.6	64	9.2
***Lifestyle habits during pregnancy***									
**Alcohol drinking** § ***********	Yes	843	27.1	168	20.3	61	9.3	103	14.6
	No	2265	72.9	351	15.5	138	7.4	198	10.2
**Drank Home Brew** ************	Yes	1723	90.2	264	15.6	105	7.3	146	9.8
	No	188	9.8	37	20.2	14	10.1	22	14.5
**Drank chibuku** ************	Yes	193	10.1	35	18.7	12	8.5	20	12.9
	No	1718	89.9	266	15.7	107	7.5	148	10.0
**Drank bottled beer** ************	Yes	47	2.5	10	21.7	5	13.2	5	13.2
	No	1864	97.5	291	15.9	114	7.4	163	10.2
**Drank wine** ************	Yes	45	2.4	3	7.0	0	0.0	3	7.9
	No	1866	97.6	298	16.2	119	7.7	165	10.3
**Smoked** *************	Yes	10	0.3	1	10.0	0	0.0	1	11.1
	No	3098	99.7	518	17.0	199	7.9	300	11.4

^§^ alcohol include: chibuku (local brew), bottled beer, spirits or wine

^†^ denotes raw percent within group, for all risk factors

* 40 observations did not have information on mother’s age

** 43 observations did not have information on maternal education

*** 201 observations did not have information on paternal education

**** 2 observations did not have information on marital status

***** 2 observations did not have information on mother’s employment status

****** 21 observations did not have information on father’s employment status

******* 14 observations did not have information on residence

******** 2 observations did not have information on electricity, water, toilet

********* 1,734 observations did not have information on BMI

********** 461 observations did not have information on mid-arm circumference

*********** 2 observations did not have information on alcohol drinking, on drinking the home brew (mahewu)

************ 1199 observations did not have information on drinking chibuku, bottled beer, wine

************* 2 observations did not have information on smoking

The mean maternal weight (and standard deviation) was 64.7(±11.4) kilograms (range = 25–135 kilograms), BMI 26.0 (±4.6) (range = 11.4–36.8) and MUAC was 27.5(±3.2) cm (range = 17.5–43 cm). In crude analysis, women with BMI ≥ 25 were 91% less likely to have a LBW, and 55% less likely to deliver a term LBW infant compared with women with BMI 18.49 to less than 25, in Table 2. Women with a BMI less than 18.49 had a 98% increased crude risk of LBW. Women with a MUAC of less than 28.5 cm compared to women with MUAC of 28.5 and more, had greater risk of delivering all three LBW categories of LBW, 75%, 2.36-fold increase, and 62% increase in the risk for all LBW, term LBW and preterm LBW infants, respectively, in Table 2.

**Table 2:** Crude Relative Risk of Low Birthweight among Live Births by Socio-Demographic Characteristics for 3110 Deliveries* at Harare Maternity Hospital, Zimbabwe.

		All LBW	Term LBW	Preterm LBW
Characteristic		Relative Risk (95% CI)**	Relative Risk (95% CI)**	Relative Risk (95% CI)**
**Total**		3110	520 (16.7%)	302 (9.7%)
***Socio-demographic factors***				
**Mother’s age**	Below 20	1.24 (0.99–1.56)	1.09 (0.79–1.51)	1.013(0.87–1.46)
	20 to 34	1.0	1.0	1.0
	above 34	0.86 (0.58–1.26)	0.69 (0.37–1.28)	1.02 (0.68–1.55)
**Maternal education**	Less than primary	1.06 (0.71–1.56)	0.89 (0.43–1.88)	1.41 (0.89–2.25)
	Primary	**0.20 (0.12–0.32)**	1.25 (0.89–1.75)	1.10 (0.83–1.47)
	Form II	1.15 (0.95–1.40)	1.23 (0.88–1.71)	1.27 (0.98–1.65)
	Form IV	1.0	1.0	1.0
	Form VI and above	0.92 (0.46–1.85)	1.01 (0.33–3.06)	0.93 (0.36–2.38)
**Paternal education**	Less than primary	1.66 (0.97–2.83)	0.82 (0.21–3.15)	1.60 (0.77–3.35)
	Primary	0.99 (0.73–1.36)	0.72 (0.39–1.34)	1.26 (0.86–1.85)
	Form II	0.97 (0.73–1.30)	0.91 (0.55–1.49)	0.92 (0.61–1.39)
	Form IV	1.0	1.0	1.0
	Form VI and above	0.95 (0.69–1.29)	0.91 (0.55–1.53)	0.75 (0.47–1.21)
**Marital status**	Current or living as married	1.0	1.0	1.0
	Never married	1.34 (0.85–2.10)	1.04 (0.40–2.69)	1.64 (0.93–2.91)
	Separated, Divorced or Widowed	**1.35 (1.01–1.81)**	1.49 (0.91–2.45)	**1.58 (1.08–2.31)**
**Mother employed**	Yes	1.0	1.0	1.0
	No	0.89 (0.72–1.11)	0.95 (0.65–1.40)	0.82 (0.62–1.09)
**Father employed**	Yes	1.0	1.0	1.0
	No	1.02 (0.83–1.26)	1.00 (0.70–1.44)	1.04 (0.78–1.38)
**Residence**	Urban	1.0	1.0	1.0
	Rural	1.19 (0.95–1.49)	**1.48 (1.04–2.11)**	1.03 (0.74–1.44)
**Have electricity**	Yes	1.0	1.0	1.0
	No	1.04 (0.88–1.24)	1.22 (0.91–1.62)	0.99 (0.78–1.26)
**Have water supply**	Yes	1.0	1.0	1.0
	No	0.99 (0.78–1.25)	1.09 (0.73–1.61)	0.97 (0.69–1.35)
**Have a toilet**	Yes	1.0	1.0	1.0
	No	0.89 (0.65–1.24)	0.58 (0.29–1.15)	1.20 (0.82–1.76)
***Anthropometric measurements***				
**Body Mass Index**	Less than 18.49	**1.98 (1.00–3.93)**	1.63 (0.44–6.12)	2.06 (0.71–5.97)
	18.49 to less than 25	1.0	1.0	1.0
	25 and above	**0.09 (0.05–0.18)**	**0.45 (0.28–0.74)**	0.70 (0.47–1.06)
**Mid-arm**	17 to 28.5 centimeters	**1.75 (1.43–2.16)**	**2.36 (1.58–3.26)**	**1.60 (1.22–2.10)**
**Circumference**	>28.5 to 43 centimeters	1.0	1.0	1.0
***Lifestyle habits during pregnancy***				
**Alcohol drinking** ***	Yes	**1.29 (1.09–1.52)**	1.26 (0.94–1.68)	**1.44 (1.15–1.80)**
	No	1.0	1.0	1.0
**Drank Home brew**	Yes	0.77 (0.57–1.05)	0.73 (0.43–1.24)	0.67 (0.44–1.02)
	No	1.0	1.0	1.0
**Drank chibuku**	Yes	1.19 (0.87–1.64)	1.13 (0.64–1.99)	1.30 (0.34–2.00)
	No	1.0	1.0	1.0
**Drank bottled beer**	Yes	1.37 (0.78–2.39)	1.77 (0.77–4.08)	1.29 (0.56–2.97)
	No	1.0	1.0	1.0
**Drank wine**	Yes	0.43(0.14–1.29)	0.36 (0.05–2.53)	0.77 (0.26–2.29)
	No	1.0	1.0	1.0
**Smoked**	Yes	0.59 (0.90–3.79)	1.40 (0.22–8.95)	0.98 (0.15–6.23)
	No	1.0	1.0	1.0

* based on data obtained from the records

** denotes 95% confidence intervals

*** alcohol include: chibuku (local brew), bottled beer, spirits or wine

During pregnancy, about 27.1% of the women drank alcohol, and most women (90.2%) reported drinking a local non-alcoholic nutritional beverage (mahewu). Alcohol drinking was associated with increased risk of LBW, and preterm LBW in the crude analysis, in Table 2. Drinking mahewu or chibuku was not associated with all the three forms of LBW reported in this study. Only 10 (0.3%) of women smoke, and smoking were not associated with LBW.

### Reproductive factors and infections

About 87% of women received prenatal care, in Table 3, and lack of prenatal care was associated with a 2.29, 1.77, 3.30-fold increases in the risk of all LBW, term LBW, and preterm LBW, respectively. Almost 86% of women were referred to Harare Maternity Hospital for delivery with 78% coming from Harare Municipal clinics. Referral was not associated with risk of all the three categories of LBW, in Table 3. Parity ranged from 0 to 9 with almost half of the women having their first child, and parity was not associated with all forms of LBW in this present day study. Having no live children was associated 18% and 43% increased risk of all LBW and term LBW, in Table 4. About 6.1% reported having a previous history of abortion, 0.3% stillbirth, 10.6% previous LBW birth. Women reporting prior history of abortion or stillbirth had a 47% increased risk of LBW and 54% increase preterm LBW in crude analysis, in Table 4. Women with prior history of delivering a LBW infant had a 70% increase in risk of all LBW, and 2.34-fold increase in delivering a preterm LBW infant. Delivering a female infant was associated with a 36%, 40% and 33% decrease in risk of delivering all three forms of LBW: all LBW, term LBW and preterm LBW, respectively, in Table 4. Therefore, male infants were less likely to be LBW, irrespective of gestational age.

**Table 3:** Distribution of Low Birthweight among Live Births by Reproductive Characteristics, and Infections for 3,110 Deliveries^§^ at Harare Maternity Hospital, Zimbabwe.

		Total births		LBW births		Term LBW		Preterm LBW	
		n	%†	n	%†	n	%†	n	%†
**Characteristic**	**Total**	**3,110**		**520**	**16.7**	199	6.4	302	9.7
**Received Prenatal Care** *	Yes	2,679	87.3	382	14.5	160	7.1	207	8.9
	**No**	390	12.7	128	33.2	31	12.6	92	29.6
**Referral status**	Yes	2,659	85.5	442	16.9	172	7.9	252	11.1
	No	451	14.5	78	17.5	27	7.7	50	13.3
**Parity** **	0	1,456	46.8	256	17.9	109	9.2	127	10.4
	**1–2**	1,243	40.0	203	16.5	73	7.3	129	12.1
	**Above 2**	410	13.2	61	15.1	17	5.1	46	12.8
**Number of children alive** **	None	1,547	49.7	281	18.5	119	9.5	142	10.9
	1 to 2	1,194	38.4	185	15.7	64	6.6	120	11.7
	**More than 2**	368	11.9	54	14.8	16	5.3	40	12.4
**History of Abortion or Stillbirth** **	Yes	199	6.4	48	24.2	16	10.4	29	17.0
	**No**	2,910	93.6	472	16.5	183	7.7	273	11.0
**History of Low Birthweight** ***	Yes	146	10.6	38	27.0	9	22.3	27	22.3
	**No**	2,543	94.6	398	15.6	167	9.5	208	9.5
Infant sex****	Male	1,588	51.2	210	13.4	79	6.0	125	9.1
	**Female**	1,511	48.8	308	20.8	120	10.0	175	13.8
***Infections***									
**Malaria during pregnancy** *****	Yes	43	1.4	13	31.0	1	3.6	10	27.0
	No	2,940	98.6	464	16.0	192	7.9	255	10.2
**Urinary tract infection** ******	Yes	74	2.6	9	12.5	2	3.3	7	10.8
	No	2,759	97.4	416	15.3	173	7.6	227	9.6
Syphilis*******	Yes	27	0.9	4	15.4	0	0.0	4	18.2
	No	1,669	57.5	217	13.2	109	7.5	101	6.9
	Not tested	1,210	41.6	235	19.7	80	8.7	143	14.4
Gonorrhea********	Yes	158	5.3	21	13.4	11	8.0	8	5.9
	No	37	1.3	27	27.0	2	7.7	8	25.0
	Not tested	2,763	93.4	437	16.1	178	7.9	243	10.3

^§^ based on data obtained from the records

^†^ denotes raw percent within group, for all risk factors

* 42 observations did not have information on prenatal care

** 1 observations did not have information on parity, number of children alive, number of previous abortions or stillbirths

*** 421 observations did not have information on history of previous low birthweight delivery

**** 11 observations did not have information on infant sex

***** 127 observations did not have information on malaria during pregnancy

****** 277 observations did not have information on urinary tract infection

******* 204 observations did not have information on syphilis during pregnancy

******** 152 observations did not have information on gonorrhea during pregnancy

**Table 4:** Crude Relative Risk of Low Birthweight among Live Births by Reproductive Characteristics and Infections, for 3,110 Deliveries* at Harare Maternity Hospital, Zimbabwe.

		All LBW	Term LBW	Preterm LBW
Characteristic		Relative Risk (95% CI)**	Relative Risk (95% CI)**	Relative Risk (95% CI)**
**Received Prenatal Care**	Yes	1.0	1.0	1.0
	No	**2.29 (1.94–2.72)**	**1.77 (1.23–2.54)**	**3.30 (2.66–4.10)**
**Referral status**	Yes	0.97 (0.78–1.20)	1.03 (0.69–1.52)	0.83 (0.62–1.10)
	No	1.0	1.0	1.0
**Parity**	0	1.08 (0.92–1.28)	1.01 (0.78–1.32)	0.86 (0.68–1.09)
	1–2	1.0	1.0	1.0
	Above 2	0.91 (0.69–1.18)	**0.56 (0.34–0.93)**	1.06 (0.78–1.46)
**Number of children alive**	None	**1.18 (1.00–1.40)**	**1.43 (1.07–1.91)**	0.94 (0.75–1.18)
	1 to 2	1.0	1.0	1.0
	More than 2	0.95 (0.71–1.25)	0.80 (0.47–1.37)	1.07 (0.76–1.49)
**History of Abortion or Stillbirth**	Yes	**1.47 (1.13–1.91)**	1.34 (0.83–2.18)	**1.54 (1.09–2.19)**
	**No**	1.0	1.0	1.0
**History of Low birthweight**	Yes	**1.70 (1.27–2.26)**	1.13 (0.59–2.14)	**2.34 (1.64–3.34)**
	No	1.0	1.0	1.0
**Infant sex**	Male	**0.64 (0.55–0.75)**	**0.60 (0.45–0.78)**	**0.66 (0.53–0.81)**
	Female	1.0	1.0	1.0
***Infections***				
**Malaria during pregnancy**	Yes	**1.93 (1.22–2.91)**	0.45 (0.07–3.10)	**2.66 (1.55–4.57)**
	No	1.0	1.0	1.0
**Urinary tract infection**	Yes	0.82 (0.44–1.51)	0.44 (0.11–1.73)	1.12 (0.55–2.28)
	No	1.0	1.0	1.0
**Syphilis**	Yes	1.16 (0.47–2.89)	0.70 (0.10–4.76)	**2.63 (1.06–6.51)**
	No	1.0	1.0	1.0
	Not tested	**1.49 (1.26–1.77)**	1.16 (0.88–1.52)	**2.08 (1.64–2.65)**
**Gonorrhea**	Yes	**0.49 (0.25–0.96)**	1.04 (0.24 -4-41)	0.24 (0.10–0.58)
	No	1.0	1.0	1.0
	Not tested	0.60 (0.35–1.02)	1.02 (0.27 -3-90)	**0.41 (0.22–0.76)**

* based on data obtained from the records

** denotes 95% confidence intervals

The frequency of mothers diagnosed with malaria during pregnancy or urinary tract infection was less than 3%, in Table 3. History of malaria was associated with a 1.93-fold increase in all LBW, and a 2.66-fold increase in preterm LBW in crude analysis, in Table 4. Urinary tract infection was not associated with all forms of LBW reported in this study. Nearly 1% (n = 27) of the women were diagnosed with syphilis, but almost half (41.6%) were not tested, in Table 3. About 5.3% women were diagnosed with gonorrhea, and were less likely to deliver a LBW infant. Women who were not tested (93.4%) for gonorrhea, were less likely to deliver a preterm LBW infant, in Table 4.

### Medical factors and obstetrical complications

The frequency of mothers diagnosed with medical or obstetrical complications, except for pregnancy induced hypertension and PROM was low, in Table 5. Less than 1% of the women had a diagnosis of anemia, diabetes, cardiovascular disease, eclampsia, or placenta Previa, and less than 10% had a diagnosis of hypertension, ante-partum hemorrhage, or history of PTL. Still, even with the small numbers, anemia was associated with a 3.51-fold increase risk of all LBW, increasing to 4.44-fold increase for term LBW and 5.15-fold increase for preterm LBW infant, in Table 6.

**Table 5:** Distribution of Low Birthweight among Live Births by Medical and Obstetrical Complications for 3,110 Deliveries^§^ at Harare Maternity Hospital, Zimbabwe.

Characteristic		Total births		LBW births		Term LBW			Preterm LBW	
		n	%†	n	%†	n	%†	n	%†
	**Total**	**3110**		**520**	**16.7**	199	6.4	302	9.7
***Medical Conditions***									
Anemia*	Yes	13	0.4	7	53.6	2	33.3	4	50.0
	No	2,886	99.6	436	15.3	179	7.5	240	9.7
Diabetes mellitus**	Yes	15	0.5	1	6.7	0	0.0	2	15.4
	No	2,752	99.5	411	15.1	170	7.4	227	9.5
Cardiovascular disease***	Yes	19	0.7	2	10.5	2	11.1	0	0.0
	No	2,743	99.3	406	15.0	168	7.3	225	9.5
Hypertension****	Yes	197	7.2	43	21.9	14	8.8	21	12.6
	No	2,560	92.8	363	14.3	155	7.2	203	9.2
***Obstetrical Complications***										
Pregnancy induced hypertension*****	Yes	678	23.4	127	18.9	57	10.4	62	11.1
	No	2,221	76.6	316	14.4	125	6.8	181	9.4
Eclampsia******	Yes	17	0.6	8	50.0	2	20.0	5	38.5
	No	2,868	99.4	436	15.4	179	7.5	240	9.7
Ante-partum hemorrhage*******	Yes	50	1.7	26	52.0	5	20.8	21	51.2
	No	2,859	98.3	430	15.2	178	7.5	234	9.5
Placenta previa********	Yes	15	0.5	5	33.3	0	0.0	6	38.5
	No	2,869	99.5	439	15.5	181	7.6	240	9.7
History of Premature rupture of membranes π*********	Yes	303	10.4	101	34.0	24	12.8	78	32.4
	No	2,609	89.6	362	14.0	159	7.2	185	8.2
History of pre-term labor#**********	Yes	230	7.9	148	64.9	13	23.2	131	75.3
	No	2,680	92.1	319	12.0	169	7.2	137	5.9

^§^ based on data obtained from the records

^†^ denotes raw percent within group

^π^ history of premature rupture of membranes with current pregnancy

^#^ history of pre-term labor with current pregnancy

* 211 observations had no information on anemia

** 328 observations had no information on diabetes mellitus

*** 333 observations had no information on cardiovascular disease

**** 338 observations had no information on hypertension

***** 196 observations had no information on pre-eclampsia

****** 210 observations had no information on eclampsia

******* 186 observations had no information on antepartum hemorrhage

******** 211 observations had no information on placenta previa

********* 183 observations had no information on premature rapture of membranes

********** 185 observations had no information on pre-term labor with current pregnancy

**Table 6:** Crude Relative Risk of Low Birthweight among Live Births by Medical and Obstetrical Complications for 3,110 Deliveries* at Harare Maternity Hospital, Zimbabwe.

		All LBW	Term LBW	Preterm LBW
Characteristic		Relative Risk (95% CI)**	Relative Risk (95% CI)**	Relative Risk (95% CI)**
***Medical Conditions***				
**Anemia**	Yes	**3.51 (2.10–5.84)**	**4.44 (1.42–13.89)**	**5.15 (2.55–10.40)**
	No	1.0	1.0	1.0
**Diabetes**	Yes	0.44 (0.07–2.94)	1.13 (0.17–7.39)	1.61 (0.45–5.81)
	No	1.0	1.0	1.0
**Cardiovascular disease**	Yes	0.70 (0.19–2.62)	1.51 (0.41–5.63)	1.17 (0.32–4.35)
	No	1.0	1.0	1.0
**Hypertension**	Yes	**1.53 (1.56–2.03)**	1.22 (0.72–5.81)	1.37 (0.90–2.09)
	No	1.0	1.0	1.0
***Obstetrical Complications***				
**Pregnancy induced hypertension**	Yes	**1.31 (1.09–1.58)**	**1.53 (1.14–2.07)**	1.18 (0.89–1.55)
	No	1.0	1.0	1.0
**Eclampsia**	Yes	**3.25 (1.98–5.35)**	2.66 (0.76–9.26)	**3.95 (1.97–9.74)**
	No	1.0	1.0	1.0
**Ante-partum hemorrhage**	Yes	**3.42 (2.58–4.53)**	**2.78 (1.26–6.15)**	**5.38 (3.89–7.43)**
	No	1.0	1.0	1.0
**Placenta previa**	Yes	**2.15 (1.05–4.43)**	1.46 (0.23–9.33)	**5.60 (3.22–9.74)**
	No	1.0	1.0	1.0
History of Premature rupture of membranes ***	Yes	**2.43 (2.02–2.92)**	**1.77 (1.19–2.65)**	**3.96 (3.15–4.97)**
	No	1.0	1.0	1.0
**History of pre-term labor** ****	Yes	**5.39 (4.69–6.21)**	**3.21 (1.95–5.28)**	**12.79 (10.65–15.38)**
	No	1.0	1.0	1.0

* based on data obtained from the records

** denotes 95% confidence intervals

*** history of premature rupture of membranes with current pregnancy

**** history of pre-term labor with current pregnancy

Hypertension had a 47% increase in the risk of all LBW infants only. On the other hand, pregnancy induced hypertension was associated with 31% increase in all LBW, and 53% increase in term LBW infants. Also, eclampsia was associated with 3.25-fold increase in all LBW, and 3.95-fold increase in preterm LBW. Ante-partum hemorrhage was associated with all the three forms of LBW, 3.42-fold, 2.78-fold and 5.38-fold increase, for all LBW, term LBW and preterm LBW, respectively. Placenta Previa was associated with 2.15 times increase in risk of all LBW, and 5.60 times increase risk of in preterm LBW. History of PROM was associated with a 2.43 times risk of all LBW, 1.77 times increased risk of term LBW and 3.96 times increased risk of preterm LBW. Also, history of PTL with a 5.39-fold increase in the risk of all LBW, 3.21-fold increase in the risk of term LBW and 12.79-fold increase in the risk of preterm LBW infants.

Table 7 presents adjusted relative risks for reproductive and nutritive factors, as well as obstetrical complications and history of infections after adjusting for relevant demographic, reproductive and nutritive factors. Maternal age and rural residence were modestly associated with all LBW, in adjusted analysis. Prenatal care, history of abortion or stillbirth, and MUMC, remained significantly associated with all LBW. Obstetric complications, except for placenta Previa, remained significant factors for all LBW in adjusted models. Malaria remained significantly associated with all LBW, while the urinary tract infection remained insignificant in adjusted analysis.

**Table 7:** Adjusted* Relative Risks for Low Birthweight^ɸ^ among Live Births, by Reproductive and Nutritive Factors^†^, Obstetrical Complications and Infections at Harare Maternity Hospital, Zimbabwe.

Characteristic	Relative Risk (95% CI)**
***Reproductive and Nutritive Factors†***	
Maternal age***	**1.01 (1.00, 1.02)**
Lack of Prenatal care	**1.69 (1.44, 1.98)**
History of Abortion or Stillbirth	**1.41 (1.18, 1.69)**
Mid-arm circumference of mother below 28.5 cm****	**1.35 (1.19, 1.54)**
Rural Residence	**1.22 (1.04, 1.40)**
***Obstetrical and Infection Complications*¶**	
Anemia	**2.63 (1.16, 5.97)**
Pregnancy induced hypertension	**1.16 (1.04, 1.30)**
Eclampsia	**2.64 (1.30, 5.35)**
Ante-partum hemorrhage	**2.39 (1.55, 3.68)**
Placenta previa	1.49 (0.71, 3.15)
History of preterm labor with current pregnancy	**4.40 (3.47, 5.57)**
History of premature rupture of membranes	**1.70 (1.44, 2.00)**
Malaria during pregnancy	**1.89 (1.21, 2.96)**
Urinary tract infection	0.91 (0.67, 1.24)

^ɸ^ All Low birthweight infants

^†^ Model 1: adjusted for referral status, whether mother was referred to Harare Maternity Hospital or not all risk factors were included in one model, estimates are adjusted for all these variables in the model

** denotes 95% confidence intervals

*** reference group maternal age 20–34 years

**** reference group MUAC ≥28.5cm

¶ Model 2: adjusted for maternal age, antenatal care attendance, drinking home brew during pregnancy and history of abortion or stillbirth

## Discussion

This paper, using a secondary data analysis, with a full data complement for over a 3-month period, estimated and examined risk factors for all LBW, term LBW and preterm LBW, among mothers delivering live births at the largest referral center in Harare, Zimbabwe. Our results suggest that the frequency of LBW is high; that nutritional factors, prenatal care, history of abortion, previous LBW, stillbirth, obstetric complications including anemia, hypertension, pregnancy induced hypertension, eclampsia, ante-partum hemorrhage are important predictors of LBW in this population. Having PTL and PROM were associated with LBW. Infection with malaria during pregnancy was also associated with LBW.

The prevalence of LBW among live-births at Harare Maternity Hospital over a three-month period of 167/1000 live births is comparable to the 168/1000 live births observed at the same hospital [10], based on delivery log data. More recently Feresu et al described a frequency rate of 19.9% and 24.3% at Harare Maternity Hospital [4, 9–12]. The prevalence of LBW for Zimbabwe from the UNICEF and WHO was 11.0% for 1999 [1, 6, 17]. Sanders et al had reported an incidence of LBW of 10.8% [9], while WHO annual reports incidence of 11.0% as late as 2013 [4, 17]. The prevalence and incidence are varying without a clear trend. There is need for more studies to depict the estimates and patterns of LBW, since it is an important contributor to infant mortality in Zimbabwe [4, 9–14].

The LBW rate in a population is a good indicator of a public health problem that includes long-term maternal malnutrition, ill health and poor health care. On an individual basis, LBW is an important predictor of newborn health and survival. Limited data from Zimbabwe suggests that LBW is a common obstetrical problem [4, 9–14], and an important contributor to infant mortality [9–11, 13–14]. Levels of infant mortality in Zimbabwe (73 per1000 live births) are high compared to South Africa (55 per 1000 live births), mid-income countries such as Mexico (25 per 1000 live births), and to developed countries including the USA, UK, or Sweden (7, 6 and 3 per 1000 live births respectively) [6–7].

We note that some conditions such as, prenatal care, infant sex, anemia, antepartum hemorrhage, PROM and history of preterm labor were associated with the three LBW categories; all LBW, term LBW and preterm LBW. Conditions like history of abortion or stillbirth, history of LBW, malaria during pregnancy, eclampsia, and placenta Previa, were associated with all LBW and preterm LBW. While, pregnancy induced hypertension, and number of children alive tend to be associated with all LBW and term LBW infants. Infections tend to be associated with preterm LBW. Chronic conditions tend to be associated with term LBW, while acute conditions tend to be associated with preterm LBW. Some risk factors or determinants are common for both term and preterm LBW. These observations may have to do with the differences in etiology for intrauterine growth restriction versus, preterm birth, although both area subset of LBW. We discuss this phenomenon, in our previous studies [10, 13, 24]. Further studies to discern these differences, will help handing of LBW in its different forms in pregnancy.

Poor nutrition measured through BMI and MUAC was adversely associated with LBW, as in previous studies [30–31], confirming the role nutrition in pregnancy [32]. Anemia was associated LBW in this population [10–11, 13], similar to other studies [33–35]. More than a quarter of mothers in our study drank alcohol, as was in previous studies [36], a modest number (10.1%) drank chibuku a local brew, while less than 3% each drank wine, or bottled beer during pregnancy. But, about 90.2% of the women drank mahewu, a nutritious drink, which in previous studies seemed to be associated with reduced risk of preterm birth [24]. In our present day study, using the same dataset, mahewu was not associated with LBW, and therefore needs further investigation.

As would be expected [10, 19, 37], lack of prenatal care was associated with LBW, and only 18.2% of women received prenatal care before 20 weeks gestation, with 10% initiating care after 37 weeks and 13% receiving no prenatal care. A comparable proportion of women who did not attend prenatal care (10.5%) was reported Feresu and colleagues [10–11, 24]. Effective interventions should dictate that women enter into prenatal programs by 20 weeks of pregnancy. Reproductive risk factors similar to other studies [18–19, 38–44], were associated with the risk of LBW. Obstetrical complications of pregnancy, although relatively infrequent, remain important risk factors for LBW in this [10–11], and other populations [38–44].

Although Harare is an urban setting, malaria appears to remain an important determinant of LBW in this population as has been shown previously [24], and in African populations [20, 45–48]. Malaria was not endemic in this setting, thus women were not likely to be screened for parasites, raising concerns about missed cases, particularly among women arriving from rural endemic areas. In Zimbabwe, screening for syphilis or gonorrhea during pregnancy is poor [24, 49], consistent with our study. Infections were not often recorded in the medical records and are not consistently screened for, thus under-diagnosis and inadequate treatment for these conditions is likely. Screening for infections such as syphilis, gonorrhea and malaria as a whole is poor, and thus we could not evaluate these important risk factors. Our present day study did not evaluate the role of HIV infection. Recent studies have tried to evaluate the role of HIV infection for perinatal outcomes including LBW and PTD, in this population, especially with the use of antiretroviral drugs [20–21, 23], however more studies are still needed to further characterize these relationships.

In this study we described the risk factors for LBW, a contributor of infant mortality, for women delivering at HMH. However, some potential limitations in our study warrant consideration. Our study focused on live-births as WHO uses this rate [1, 3, 6], but excluding stillbirth and early neonatal deaths underestimates the true burden of LBW and may have biased our results towards the null hypothesis. Focusing solely on births within Harare Maternity Unit raises concerns about potential selection bias, however, the risk of LBW did not differ between those referred and not referred, and referral status was controlled for in the final model on demographic, nutritive and reproductive factors. Estimates of LBW are within range of other studies [1, 9–12, 17]. Exclusion of twin deliveries and stillbirth somewhat underestimates the rate of LBW at this hospital. Prenatal care in this study was defined as attending care irrespective of gestational age. We did not have proper estimates of timing and number of visits. Lack of adequate data on the number of prenatal care visits limits our ability to assess the adequacy of prenatal care. Our study relied on medical records, raising concerns of incomplete data. A culmination of poor reporting, lack of adequate screening for medical conditions, and infections, and ensuing possibility of missed exposed cases, raises concerns about non-random misclassification.

Our small sample size did not allow us to further explore term, term LBW, and preterm LBW birth relationships with risk factors in adjusted analysis, nor could we analyze for very low and extremely birthweight infants. Albeit, we were also able to examine crude risks for term and preterm LBW infants, a distinction that would be important for maternity care in this population. Importantly, we have shown that traditionally established risk factors including nutrition, prenatal care, maternal risk factors, medical conditions, obstetric complications and malaria remain important risk factors for LBW in this urban Zimbabwean population.

## Conclusion

Our study is among few studies evaluating risk factors for LBW in Zimbabwe. We have demonstrated similar and different risk factors for subsets of LBW. This step is important for targeted interventions. Programs aimed at improving women’s health focused on improving nutritional status of women remain of critical importance for this population. Perhaps most importantly, adequate and focused screening and evaluation of the role of infections, such as malaria, syphilis, and HIV comorbidity, shown to be a risk factor for LBW in previous studies [20–21, 23, 50], is warranted. More studies are imperative for Zimbabwe, as more prevention efforts are needed, if the goal is to ultimately reduce infant mortality and morbidity.
